# Physical activity interventions in African American women: A systematic review

**DOI:** 10.15171/hpp.2017.11

**Published:** 2017-03-05

**Authors:** Vanessa Bland, Manoj Sharma

**Affiliations:** Behavioral & Environmental Health, School of Public Health, Jackson State University, MS, USA

**Keywords:** Exercise, Physical activity, Blacks, African Americans, Women

## Abstract

**Background:** African American women are at high risk of acquiring chronic diseases due to sedentary lifestyles. This objective of this article was to perform a narrative systematic review of physical activity interventions among African American women published between 2009 and 2015.

**Methods:** A review of literature in following databases: Academic Search Premier, CINAHL (Cumulative Index to Nursing & Allied Health), ERIC (Education Resources Information Center), MEDLINE, PsychInfo, and SPORTDiscus was performed to locate interventions promoting physical activity among African American women.

**Results:** The search yielded 13 interventions. All the studies were conducted within the United States. It was found that walking coupled with healthy food choices were salient strategies in the interventions. Studies using social support along with healthy diet were found to be more efficacious in fostering physical activity among African American women.

**Conclusion:** Walking, social support and a healthy diet were found to be significant strategies promoting physical activity in African American women. Physical activity for African American women must build on the constructs of healthier food choices and social support.

## Introduction

Physical activity comprises of any bodily movement produced by skeletal muscles that use energy. Physical inactivity has been recognized as the fourth leading risk factor for mortality around the world causing an estimated 3.2 million deaths.^1^ The US President’s Council on Physical Fitness and Sports formed in 1956 underscored the importance of physical activity for a long time. Since 1990, in United States every 10 years *Healthy People Objectives* have been published that have included objectives for increasing physical activity.^2^ The World Health Organization (WHO)^3^ also recommends that adults between the ages of 18–64 years must participate in at least 150 minutes of moderate-intensity aerobic physical activity during the week or at least 75 minutes of vigorous-intensity aerobic physical activity during the week.

Despite the recommendations there are several subsections of the society that do not engage in sufficient and regular physical activity. African American women are a particular susceptible group for unhealthy lifestyles, particularly physical inactivity, and the ensuing chronic diseases and outcomes.^4^ Obesity has increased in African American women in United States with obesity being around 50%.^5^ Obesity poses a risk for several adverse health outcomes such as heart disease, type 2 diabetes, obstructive sleep apnea, and other diseases.^5^

The prevalence of obesity is significantly greater among African American women as compared to the Caucasian American counterparts.^6^ Further, African American women are more likely to underestimate their body weight which is associated with reduced weight management behaviors, higher weight gain and an under assessment of health risks. This under estimation of weight can be an important barrier to effective weight management in African American women.^6^ Perception of body image can be an indicator of health risks and weight status and is associated with race. As mentioned earlier, Black women often misjudge their body size and do not report body image dissatisfaction in comparison to Whites.^7^ Furthermore, as mentioned earlier Black women have higher rates of sedentary behavior compared to other racial/ethnic subgroups. Consequently, African American women have higher morbidity and mortality associated with inadequate levels of physical activity and sedentary behavior. Hence, effective strategies to enhance and sustain physical activity among this at-risk population are critical.^8^ Physical activity promotes a healthier lifestyle and reduces the risk of chronic diseases and premature death.

Previous research has found that there are cultural differences regarding physical activity between African American and other races. There is a commonality of lack of time, deficient energy, and time devoted to caregiving by all women for not being physical active. However, African American women have their unique reasons for not being active such as lack of a safe place to be physically active, being self-conscious and not being overtly conscious of their weight and body size.^9^

Black community in general is more religious than other groups with 79% mentioning church being very important in their lives.^10^ Interventions that utilize church as a setting have a greater potential for reaching out African Americans and facilitating regular physical activity in this community.^10^ Sharma et al^11^ conducted a cross-sectional study to examine the degree to which self-efficacy for physical activity and social support were related to the length of time for leisure-time physical activity in a group of African American women. The study found that self-efficacy for moderate-intensity leisure-time physical activity and frequency of friends’ social support were significant predictors constructs.

There have been interventions that have been designed for African American women. However, in recent years no systematic review has been done on interventions pertaining to African American women. Such a review can identify what works and what does not work and provide guidance to future interventions.

It is in this context that the objective of this paper is to provide a narrative and systematic review of physical activity programs among African American women published between 2009 and 2015. For the sake of this paper, the terms African American and Black has been used interchangeably. The primary focus of this paper is to discuss whether the interventions increased physical activity among African American women. Duration, frequency, intensity levels, tracking of the interventions and how the interventions are prescribed were the key aspects that were reviewed.

## Materials and Methods

A review of literature was performed to locate interventions for physical activity published between 2009 and 2015 among African American women in following databases: Academic Search Premier, CINAHL (Cumulative Index to Nursing & Allied Health), ERIC (Education Resources Information Center) MEDLINE, PsychInfo, and SPORTDiscus. The first search used the keywords “physical activity interventions” and “African American women”; the second search used the keywords “physical activity interventions” and “Black women” and; the third search used the key words “physical activity interventions,” “African American/Black women” and “randomized control trials”; the final search used the keywords “physical activity interventions,” or “physical activity programs” and “African American/Black women.”

**Figure 1:**
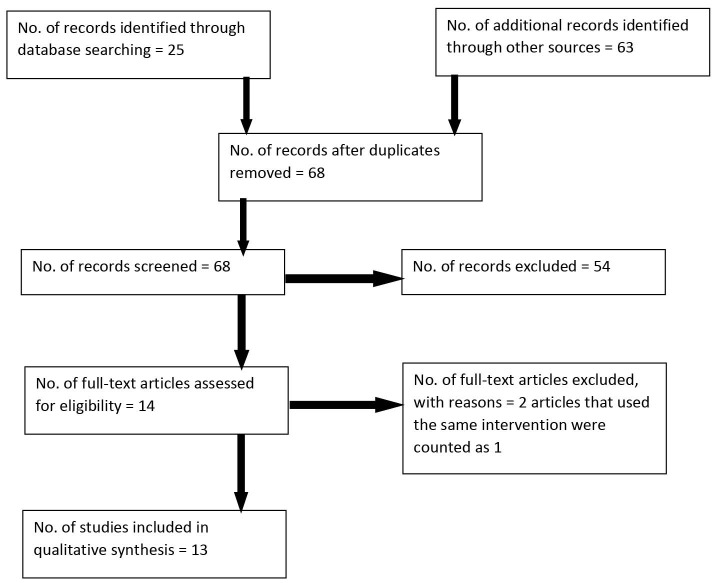
Intervention article selection process based on PRISMA guidelines.

The inclusion criteria were articles (1) that included African American women (18 years and older), (2) publications in English language, (3) publications between January 2009 and January 2015, (4) full-text/scholarly peer-reviewed journals (5) that included evaluation of interventions as opposed to mere description and (6) that included both qualitative and quantitative and impact evaluations. Articles were excluded from this review based on the following: (1) if the articles were identified as review articles, (2) articles written in languages other than English, (3) articles than included ethnicities other than African Americans and (4) dissertations. This is further illustrated in Figure 1. Preferred Reporting Items for Systematic Reviews and Meta-Analyses (PRISMA) guidelines were used in retrieving the articles,^12^ There were two researchers who conducted the search. One was a doctoral student of public health and another one was a professor of public health aided by a librarian. The data were searched from the databases and the title and abstracts were read to see if they met the inclusion criteria. When in doubt the full text article was obtained. The search was confined to the published literature in the six databases and articles from the grey literature were not included in the search. The data used was from published articles only and the authors were not contacted for any clarifications. The data were extracted in the form of a Table that is presented in this review.

## Results

The data extraction method yielded a total of 14 studies evaluating 13 distinct interventions from 2009 to 2015 that met the inclusion criteria, which is presented in Table 1. Table 1 presents a summary of the studies’ important attributes that included physical activity interventions in African American women. The 13 interventions included focused on African American women studies conducted in the states of Alabama, Mississippi, Arizona, California, Illinois, North Carolina and South Carolina. Sample sizes of these studies ranged from 15 to 565 participants and the means of reported means of the sample size were 155.64 with a standard deviation of 173.53. The studies included participants ranging from 18 to 70 years of age among those studies that mentioned the participants age. The reported means of age were 48.28 years with a standard deviation of 10.85 years. Of the 13 interventions, with the combination of the two articles that used the same intervention,^13,14^ four of the studies’ were conducted at community health centers,^15-18^ three of the studies used several churches as their study setting,^19-21^ two were conducted in southern rural communities,^13,14^ two used a medical center as the setting of choice,^22,23^ one used two beauty salons,^24^ one used a college university location^25^ and one used several counties in the Alabama area as the study setting.^26^

Majority of the studies (n=6)^16,17,19,21,22,25^ were randomized controlled designs. Three of the studies used quasi-experimental designs^16,18,24^ and one used a qualitative exploratory design.^15^ The remaining studies were pilot and feasibility studies. Additionally, one of the studies used both a quasi-experimental and randomized controlled design.^16^ Many studies used behavioral theories in their theoretical framework to explain, predict or show relationship between the studies’ research hypothesis and problems.

The duration of the physical activity interventions varied from 5 weeks to 24 months with a mean of 34.07 weeks and a standard deviation of 31.48 weeks. The studies’ physical interventions included walking and running exercises, indoor/home activities (cooking, cleaning, dusting, gardening), line dancing, praise dancing, yoga, Zumba, kickboxing and aerobics. The studies’ either included a physical activity intervention(s) and/or utilized physical activity questionnaires to ask participants what physical exercises they were incorporating into their daily routine. The frequency of the physical activity interventions ranged from 15-30 minutes per day, 1-9 hours per week or 3-5 times a week. The physical activity interventions were tracked by pedometers, accelerometers, heart rate monitors, automated telephone system, walking log books and diaries. Majority of the interventions prescribed were moderate to vigorous intensity exercises. The interventions were prescribed by means of group counseling sessions, telephone coaching/counseling, workshops, focus groups and educational sessions/meetings.

Physiological data were measured by 7 interventions included blood pressure,^13,14,17,19,20,22^ capillary blood flow,^17^ heart rate,^16,20^ fasting lipid panel that included total cholesterol, high-density lipoprotein (HDL-C) and low-density lipoprotein (LDL-C)^1,3,14,20,22^ triglycerides,^14^ glucose levels^14^ and waist circumference.^13,14,17^ Common primary outcomes measured were diet,^17,24,25^ fried food consumption,^25^ water consumption,^25^ barriers to physical activity,^15,22,23^ physical activity adherence,^13,15,16,18,23^ neighborhood characteristics^16,18^ and spirituality.^21^ Psychological well-being^18,21^ and social support^13-15,20,22,24^ was also evaluated in some of the studies. Physical activity was assessed by the following scales, questionnaires and surveys: Community Health Activities Model Program for Seniors (CHAMPS),^17,19^ Behavior Risk Factor Surveillance Survey,^26^ Physical Activity and Disability Survey (PADS).^22,23^ Barriers to Physical Activity and Disability Survey (B-PADS)^22,23^ were used to asses environmental and facility barriers and the social support for physical activity were measured by the Social Support Questionnaire (SSQ)^20^ which measured the specific domains of social support including appraisal, belonging, tangible and self-esteem. The New Leaf Dietary Risk Assessment (DRA)^17^ were used to evaluate diet, the Center for Epidemiological Studies-Depression (CES-D) scale^18^ were used to assess depressive symptoms and the Quality of Well-Being (QWB) scale^22^ assessed expression of well-being. In addition, global questions (“How many minutes of physical activity [walking, running, or other exercise] do you get daily?” were also used to measure physical activity that were developed form a pre-and posttest questionnaire.^24^

Instruments commonly utilized in the studies to measure physical activity were pedometers,^13,14,19,21,22,25^ accelerometers^20,21^ and heart rate monitors.^15,16,18,25^ Focused groups, interviewing, and telephone-based counseling were techniques used to connect, support and motivate the women’s progress to encourage healthy behaviors. Also, these techniques were used as a strategy to collect data in relation to prior level of physical activity behavior, expected outcomes and perceived outcomes of the participants experience regarding compliance and adherence to physical activity.

## Discussion

Physical inactivity is a public health concern. The development of physical activity interventions is important for those at risk of developing other health conditions. The purposes of this review were to look at studies published between 2009 and 2015 to provide a narrative and systematic review of interventions for physical activity conducted among African American women. A total of 13 interventions met the inclusion criteria.

From the 13 interventions, seven of the physical activity programs demonstrated that the interventions increased physical activity among African American women^13,14,17,19,20,22,23^ and two of the studies showed that there was an increase intake of fruits and vegetables.^24,26^ Although, the interventions demonstrated positive changes in physical activity, a few of the studies^17,23^ still presented barriers to physical activity. Two studies demonstrated that physical activity was not sustained throughout the 24-month period,^26^ nor at post-test the findings were significant for water consumption and physical activity.^24^ The physical activity interventions demonstrated that limiting barriers to adhere to physical activity and safe neighborhoods are significant in the increase of physical activity among African American women.

Of the 13 interventions, only seven used randomized controlled trials.^16,17,18,19,22,26^ Randomized controlled trials are considered the gold standard for clinical purposes. More studies must utilize randomized controlled designs so that more evidence-based literature can be generated.

Social cognitive theory and the trans-theoretical model were commonly used approaches utilized in the studies. Social cognitive theory premises that there is an interaction of personal factors and environment on behaviors including sustenance of physical activity.^21^ Theories are useful in measuring behavioral concepts and/or reasons for certain behavioral patterns. Sample size was found to be a limitation in some of the studies. The studies should focus on an adequate number of participants to generalize the results to a large number of individuals otherwise a small sample size lacks power of the statistical results. Another noted limitation was that a few of the physical activity interventions showed no significance after follow-up. This suggests that further research is needed to develop interventions that will increase physical activity among African American women.

Physical activity interventions should target habits and personal behaviors that limit physical activity. Physical activity interventions primary concern should not be directed toward weight loss because there are other areas that affect physical activity that many researchers do not address. Researchers should incorporate behavioral components in physical activity interventions to understand the perceptions of the lack of physical activity among African American women.

The review had several limitations. First, the identified physical activity interventions had the time frame of only 2009 to 2015. Although, more current information is typical in research, excluding studies outside of the time period may cause the researcher(s) to disregard significant information from previous studies that may be favorable to a present and/or future study.

Secondly, the study was restricted to specific databases. Only six databases were used to obtain information on this topic. This omits other databases that may provide important information pertaining to the topic. And third, the physical activity interventions chosen were of English language. Foreign interventions were excluded granting such interventions also provide important information regarding physical activity in children and adults targeting an increase in physical activity. Despite the limitations of the study, the findings do support previous research demonstrating that educational physical activity interventions do have a positive influence to enhance physical activity for African American women.

## Implications for future studies

Physical activity interventions should not primarily target weight loss but healthy dietary habits, changes in behavior and the environment. Lack of social support and countless responsibilities are reasons for the lack of physical activity among this group of women.^19^ One way to address this issue is by providing neighborhood facilities that provides support for women that desire to have a more active lifestyle. Incorporating nutritional classes during fitness hours may also be helpful to address unhealthy dietary habits.

To address changes in behavior, physical activity interventions may consider incorporating behavioral theories. The intervention itself will not change the individuals’ behavior; rather the implemented activities that target certain behavioral patterns that influences behaviors. Therefore, such interventions should be measured and tested to establish valid and reliable results. This will provide evidence that theory-based interventions will exemplify power among certain target groups.

Finally, researchers and public health professionals should consider the built environment when developing physical activity interventions. Researchers should consider the geographical features of given neighborhoods to determine ways to modify the environment to increase physical activity in all areas. This may propose developments of physical activity policies and/or recommendations for future research interventions.

## Conclusion

Physical inactivity is a major public health issue. Due to the lack of physical activity, there has been an increase in obesity, diabetes and cardiovascular disease. More groups are affected by these diseases and the lack of physical activity, particularly African American women. One way to address this issue is to develop interventions that increase physical activity among African American women. Of the total 13 interventions, there was an increase in physical activity in 7 of the physical activity interventions. This shows that African American women can adhere to physical activity interventions. In all, the studies’ were associated with one another because the aim of the studies’ were to provide information regarding interventions for African American women and/or provide useful information that researchers my utilized to develop other interventions to promote physical activity among African American women. Despite the limitations of the studies, the interventions implemented did show an increase in physical activity.

## Ethical approval

Ethical considerations are not applicable for this paper.

## Competing interests

The authors have no conflicts of interest to report.

## Authors’ contributions

VB conceptualized the study, collected the articles, summarized the articles and prepared the first draft of the manuscript. MS helped in conceptualizing the study, collected the articles, and helped in finalization of the manuscript.

## Disclaimer

The authors claim that no part of this manuscript has been copied from other sources.

## Acknowledgements

The authors are thankful to Jackson State University for its support.

**Table 1:** Summary of physical activity interventions in African American women

**Article reference**	**Age of subjects and** **No. of participants in study**	**Study setting**	**Theory**	**Study design**	**Intervention description**	**Key findings**
Rimmer et al^22^	Age: 18+ years of age n=92	University of Illinois at Chicago Medical Center	Not specified	RCT	Awareness group: information brochure on physical activity and a toolkit Lower support group: weekly telephone consultation, toolkit and monthly newsletter Higher support group: same intervention as lower support group plus participation in a monthly onsite exercise support group	* Both the higher and lower support groups demonstrated significant increases in physical activity scores (39% and 30% respectively, *P*<0.05 for both pre-post comparisons) during the intervention phases
Wilbur et al^18^	Average age: 48.5 years of age n=278	Chicago community health centers	Social cognitive theory & transtheoretical model	Quasi-experimental design	Women’s Walking Program: 24-week, home-based, moderate-intensity walking intervention	* There were no significant differences between the treatment groups (enhanced treatment and minimum treatment) on any of the base line individual or neighborhood characteristics.
Zoellner et al^14^Zoellner et al^15^	Age: average age of 44 years n=83	Hollandale, MS	Social support and transtheoretical model	Feasibility study	Participants were asked to record, at the end of each day, the time the pedometer was put on, the time the pedometer was taken off, and the total number of daily steps and to circle whether they walked alone or with others.	*Participants increased their steps by approximately 39% or 2600 steps per day over the 6-month intervention and reported higher percent increases in the beginning months of the intervention
Johnson et al^24^	Age: 18-70 n=20	Two beauty salons in South Carolina	Not specified	Quasi-experimental design Pilot study	Steps for a New You intervention The motivational sessions consisted of three parts during a 6-week period and provided clients with encouragement to begin to adopt healthy behaviors, using the cosmetologist as the health promoter.	*With regard to physical activity, the mean daily minutes changed little between pretest and post-test for the treatment group. *There was an increases in the comparison group between pretest and post-test but the change was not significant
Duru et al^19^	Age: >60 years of age n=62	3 Los Angeles Churches	Not specified	RCT	The 8-week intervention curriculum was designed to incorporate evidence-based best practice approaches for physical activity programs targeting older adults	*At 6-month follow-up, intervention participants increased their mean weekly walking activity by 7457 steps more than control participants, on average (p=0.016).
Oh et al^17^	Age: 40-65 n=148	Two community health centers in the city of Chicago	Social cognitive theory & transtheoretical model	RCT Quasi-experimental design	Both groups (minimal treatment [MT] and enhanced treatment [ET]) received an orientation to walking and stretching techniques The ET group received 4 weekly motivational workshops followed by tailored supportive staff telephone calls	*On average, participants completed 36.9% (standard deviation [SD] =33.5) of prescribed walks including 43.3% (standard deviation = 34.0) for the enhanced treatment group and 26.2% (SD = 28.1) for the minimum treatment group.
Peterson et al^20^	Age: 35-65 years of age n=18	Church	Social comparison theory	Feasibility study	The HSPAP is based on appraisal, belonging, tangible, and self-esteem domains of social support	*The total minutes of physical activity per week reported on the 7-DAR increased significantly, *t*(17) = 2.29, *P*<0.05, during the 6-week study from a mean of 412 min per week (SD = 100) at baseline to a mean of 552 min per week within 6 weeks (SD = 246).
Rimmer et al^23^	Age: 45-64 years of age n=33	Referred by primary care physician from an internal medicine clinic of a Mid-western university medical center	Not specified	Pilot study	6-month telephone-based physical activity coaching intervention: weekly calls between 15-30 minutes to assist the participant in identifying the barriers to physical activity	*There was a significant increase in total minutes per day of structured exercise (*t* [32] = 4.05, *P*=0.00), general indoor household physical activity (*t* [32] = 2.06, *P*=0.048, and total physical (*t* [32] =3.94, *P*=0.00).
Ingram et al^16^	Age: 40-69 years of age n=281	Community health centers in Chicago		Qualitative exploratory design	Women’s Walking Program: a home-based 12 month community based intervention that included orientation, focus group workshops, telephone contacts and walking prescriptions	*The primary barriers reported by both low adherers and high adherers were limitations related to family and work responsibilities, weather, and neighborhood safety.
Parra-Medina et al^27^	Age: 35+ years of age n=51	9 community clinics within 2 community health centers in South Carolina	Transtheoretical model & social cognitive theory	RCT	Heart Healthy and Ethically Relevant (HHER) Lifestyle trial assessed the effectiveness of a culturally appropriate, theory-based intervention delivered in primary health care settings to reduce dietary fat and increase moderate-to-vigorous physical activity among financially disadvantage African American women.	*Comprehensive intervention participants were significantly more likely than were those in standard care to decline in total physical activity at 6 months (adjusted odds ratio [OR] = 3.13; 95% confidence interval [CI] = 1.18, 8.25) *They were significantly more likely to improve in leisure-time physical activity (adjusted OR = 3.82; 95% CI = 1.41, 10.3).
Whitt-Glover et al^8^	Age: >18 years of age Number of participants not specified	North Carolina 30 churches	Social ecological and social cognitive theory	Cluster randomized control trial	Cluster 1 received a faith-based intervention curriculum that combined behavior change and social learning theories with spiritual tenets. Cluster 2 received a traditional non-faith-based intervention curriculum based on principles of behavior change and social learning theories to increase physical activity. Cluster 3 received standard written materials to increase physical activity.	*Data from the pilot study showed that average increases in daily steps were 1013 at week 12 and 1521 at week 24 with standard deviations of 1584 and 2524.
Joseph et al^25^	Age: 19-30 years of age n=15	Undergraduate and graduate college females at a University Arizona State University University of Alabama at Birmingham	Not specified	Pilot study	For two weekly session, participants walked the indoor track of the university’s recreation center at a moderate intensity For the other two sessions, participants exercised on their own or participated in a cardiovascular-based group exercise class	*BMI significantly decreased over the duration of the study (*P*=0.034), reflected by a marginal decrease in body weight (*P*=0.057).
Scarinci et al^26^	45-65 n=565	6 counties in the Alabama Black Belt	Not specified	RCT	Intervention arm: 5-week healthy lifestyle intervention Comparison arm: educational and behavioral strategies to promote breast and cervical cancer screening	*There as a significant change in physical activity between arms (*P*=0.004), but the change in physical activity was not associated with any other factors.
